# 

**Published:** 1907-05-15

**Authors:** 


### ANNOUNCEMENTS.

JAMESTOWN EXPOSITION, NORFOLK, VIRGINIA, 1907.-- THE JAMESTOWN DENTAL CONVENTION, NORFOLK, VIRGINIA, September 10–12th, 1907.

COMMITTEE ON ORGANIZATION.

Burton Lee Thorpe, Chair., St. Louis, Mo.

H. Wood Campbell, Sec. Suffolk, Va.

R. H. Walffer, Norfolk, Va.

F. W. Stiff, Treas., Richmond, Va.

Thos. P. Hinman, Atlanta, Ga.

J. E. Chase, Ocala, Fla.

Clarence J. Grieves, Baltimore, Md.

The Jamestown Dental Convention will be held in an especially equipped building on the Exposition Grounds, which was built for the purpose of accommodating this convention, the building is equipped with an auditorum, committee rooms, and excellent facilities for conducting dental clinics and for holding exhibits. All of these will be held in this building. The entrance to the building is outside of the grounds. However, one may obtain access to the grounds through it. The building is wired with both direct and alternating current, equipped with running water, well lighted and contains all modern conveniences, thus making it an ideal convention hall. The exhibits are under the management of Dr. John W. Manning, Bank of Commerce Bldg., Norfolk, Virginia. To him exhibitors should apply at once for space. Price per foot and a plat of the hall will be sent upon request. The clinics are under the supervision and direct control of Dr. C. J. Grieves, Park and Madison Aves.,

Baltimore, Md. His assistants are Drs. Baskerville Bridgeforth, Richmond, Virginia, E. J. Tucker, Roxboro, N. C. Herbert Johnson, Macon, Ga., F. A. Bowles, Washington, D. C. and Joseph T. Meaders, Nashville, Tenn. The prospects are that the Jamestown clinic will be the largest and most complete dental clinic ever held. Assistants clinic chairmen have been appointed in each State in the Union and nearby countries, viz: Canada, Mexico, Cuba and Hawaii. From these come reports of the enlistment of the best clinic talent in their respective States and Countries.

Membership chairmen have been appointed in the various States and Countries. Names of these and the clinic chairmen will appear with the list of other officers in this issue of this journal. The membership commttee is headed by Dr. F. W. Stiff, the general chairman, 600 East Grace St., Richmond, Va., who already reports memberships rapidly coming in. The Hotel head-quarters will be at the Inside Inn, where reasonable rates and excellent accommodations are assured. The Inside Inn generously offers numerous halls and committee rooms free of charge to the various college fraternities and alumni, who are invited to hold thier meetings in these rooms. Later reports as to Hotel accommodations and prices will appear in a subsequent issue of this journal. The Membership fee is five dollars, which will entitle members to a bound copy of the proceedings. A half rate $2.50 is made to bona fide dental students upon certificates from the Deans of their colleges, and when presented to the state chairman of the membership committee, for endorsement and acceptance, will entitle them to the rights and privileges of the convention. The essayists are to be, Prof. W. D. Miller, of Ann Arbor, Mich; Dr. F. T. Van Woert, Brooklyn, N. Y.; Dr. Chas. L. Alexander, of Charlotte, N. C.; D. R. Ottolengui, of N. Y. Dr. E. P. Beadles was elected by the Committee on Organization, in Feb. to go to Europe and extend a cordial invitation to dental societies and individual dentists to attend the Convention. The following officers were elected by the Committee on Organization at its recent meeting Feb., 23rd, 1907.

Honary President, Dr. J. Y. Crawford, Nashville, Tenn.; President, Dr. V. E. Turner, Raleigh, N. C.; First Vice President, B. Holly Smith, Baltimore, Md.; Sec'y-General, Dr. Geo. F. Weesee, Richmond, Va.; Treasurer, Dr. Mark F. Finley, Washington, D. C.; Vice Presidents, Drs. W. D. Miller, Geo. L. Field, Michigan; A. A. McClanahan, T. J. McClanahan, F. A. Shotwell, G. Noel, A. S. Mellendy, Tennessee;Dr. A. C. McCurdy, Maryland; Drs. Edward Eggleston, W. E. Norris, Virginia; Drs. D. N. Rust, W. W. Evans, Geo. W. Boynton, Washington, D. C.; Drs. J. R. Osborne, C. L. Alexander, North Carolina; Dr. S. H. Johns, Wilmington, Del.; Dr. Max M. Eble, Louisville, Ky.; Drs. Chas. L. Gonn, G. S. Van, J. A. Hall, Alabama; Drs. R. W. Quarles, T. M. Milan, Van Buren, Ark.; Drs. Wm. Crenshaw, H. H. Johnson,Georgia; Drs. S. F. Kemp, W. G. Mason, L. C. Elkins, Florida; Drs. J. H. E. Milhous, T. T. Moore, South Carolina; Dr. L. B. McLaurin, Mississippi; Drs. F. A. Blanchard, J. E. Woodward, Louisiana; Drs. R. D. Griffis, P. S. Turner. J. W. David, Texas; Dr. F. T. Van Woert, Brooklyn, N. Y.; Drs. J. D. Patterson, D. O. M. LeCron, W. G, Dalrymple, Missouri; Drs. Wm. M. Bebb, Garrett Newirk, California; Dr. James McManus, Conn. Dr. Geo. Edmond Hunt, Indiana; Drs. G. V. Black, T. W. Brophy, A. H. Peck, Illinois; Drs. G. M. Work, D. J. McMillan, Alton H. Thompson, Kansas; Drs. R. R. Andrews, W. E. Barden, G. E. Mitchell, Massachusetts; Drs. E. K. Wedlestadt, A. C. Searl, Minnesota; Drs. T. M. Hampton, Geo. Longway, Montana; Drs. H. F. King, Edwin C. Bladsell, New Hampshire; Drs. Chas. A. Meeker, R. M. Sanger, New Jersey; Drs. H. J. Burkhart, Ottolengui, Wm. Carr, New York; Drs. Geo. H. Wilson, L. C. Custer, Ohio; Drs. Norris R. Cox, Arthur W. Chance, Oregon; Drs. Edward C. Kirk, Wilber F. Litch, Edwin T. Darby, H. C. Register, Pennsylvania; Dr. W. Leon Ellerbeck, Utah; Dr. Chas. F. Irwin, Washington: Drs. G. V. I. Brown, W. A. Cudworth, Wisconsin; Dr. A. J. Derby, Hawaii, Honolulu; Dr. Andres C. Weber, Havana, Cuba, Corelas; Drs. Falero Mexico, Richardo Figueroa, Mexico.

### General Clinic Committee.

Clarence J. Grieves, Chairman, Park & Madison Aves., Baltimore, Md.; Baskerville Bridgeforth, Richmond, Va.; E. J. Tucker, Roxboro, N. C.; H. Herbert Johnson, Macon, Ga.; F. A. Bowles, Washington, D. C.; Joseph T. Meadors, 625 Church St., Nashville, Tenn.

### State Chairman of Clinics.

Alabama—L. A. Crumley, Hood Bldg., Birmingham; Arkansas—Chas. Richardson, Fayetteville; California—Frank L. Platt, 712 Steiner St., San Francisco; Connecticut—Chas. McMannus, 80 Pratt St., Hartford; Colorado—W. E. Sinton, El Paso Bldg., Colorado Springs; Deleware—C. R. Jeffris, New Century Bldg., Wilmington; Dist. of Columbia—H. J. Allen, 303-4 Colorado Bldg., Washington; Florida—Carroll H. Frink, Fernandina, Georgia—A. M. Jackson, Macon; Idaho—J. B. Burns, Fayette; Indian Territory—S. F. Long, South McAllister; Indiana—Carl D. Lucas, Willoughby Bldg., Indianapolis; Iowa—C. M. Work, Ottumwa; Kansas—Frank O. Hetrick, Ottawa; Kentucky—E. D. Rose, Bowling Green; Louisiana—Joules J. Sarrizan, New Orleans; Maine—H. A. Kelley, 609 Congress St., Portland; Maryland—George E. Hardy, Baltimore; Michigan—E. B. Spalding, 4 Adams Ave., West Detroit; Massachusetts—C. W. Rodgers, Dorchester; Minnesota—J. W. S. Gallagher, Winona; Mississippi—W. R. Wright, Jackson; Missouri—E, P. Dameron, De Meniel Bldg., St. Louis; Montana—G. E. Longway, Great Falls; Nebraska—H. A. Shannon, Lincoln; Nevada—J. C. Hennessy, Reno; New Hampshire—John W. Worthen, Concord— New York—Dwight Tracey, 46 W. 51st St., New York City; New Jersey—G. W. E. Holbrook, 2 Say Brook

Place, Newark; North Carolina—J. A. Corran, Asheville; North Dakota—C. L. Rose, Fargo; Ohio—H. C. Brown, 185 E. State St., Columbus; Oklahoma—Theodore P. Brinkhurst, Shawnee; Oregon—Arthur W. Chance, DeKum Bldg., Portland; Pennsylvania— H. B. McFadden, 3505 Hamilton Ave., Philadelphia; Rhode Island—Dennis F. Keefe, 315 Butler Exchange, Providence; South Carolina—Thomas T. Moore, Jr., Columbia; South Dakota—E. S. O'Neil, Canton; Tennessee—A. J. Cottrell, Knoxville; Texas—John W. David,Corsicana; Utah—William Leon Ellerbeck, 21 Hooper Bldg., Salt Lake City; Vermont—E. O. Blanchard, Randolph: Virginia—R. L. Simpson, Richmond; Washington—C. A. Custer, Chagin Block, Seattle; West Virginia—F. L. Wright, Wheeling; Wisconsin—W. A. Cudworth, Milwaukee; Mexico—J. Falero, 18 Tacuba, City of Mexico, Cuba—Andres G. Weber, Corelas 1 Esq a Egido, Havanna; Hawaii—A. J. Derby, Honolulu.

### General Membership Committee.

F. W. Stiff, Chairman 600 East Grace St., Richmond; A. S. Melindy, Knoxville, Tenn.; William Crenshaw, Atlanta, Ga.; M. S. Merchant, Houston, Texas, Mason, Bldg.

### State Chairman for Membership

Alabama—C. S. Gunn, Gadsden; Arkansas—T. M. Milam, Little Rock, Mann Bldg.; California—J. Lorenz Pease, Oakland; Connecticut—Frederick T. Murless, Jr., Windsor Locks; Colorado—Henry F. Hoffman, 612 California Bldg., Denver; Delaware—S. H. Johns, Wilmington; Dist. of Columbia—William N. Cogan, Washington; Florida—F. E. Buck, Jacksonville; Georgia—Walter G. Miller, Agusta; Idaho—J. H. Lewis, Nez Perce; Illinois—Frederick B. Noyes, Stewart Bldg., Chicago; Indiana—Frederick R. Henshaw, Middletown; Indian Territory—J. M. Staples. Atoka; Iowa—F. T. Breene, Iowa City; Kansas—F. G. Corey, Council Grove;

Kentucky—A. B. Dixion, Glasgow; Louisiana—G. Victor Vinges, Macheca Bldg., New Orleans; Maine—Will S. Payson, Castine; Maryland—W. G. Foster, 9 West Franklin St., Baltimore; Massachusetts—Waldo E. Boardman, 419 Boylestou St., Boston; Michigan—Albert L. Le Gro, 271 Woodward Ave., Detroit; Minnesota—James Elmer Weirick, St. Paul; Mississippi—A. E. Tillman, Vicksburg; Missouri—D. O. M. Le Cron, Mo. Trust Bldg., St. Louis; Montana—T. M. Hampton, Helena; New Jersey—Alphonse Irwin, Camden; Nebraska—E. H. Bruening, Omaha; New Hampshire—H. P. Baldwin, Manchester; New York—H. Clay Ferris, 1166 Dean St., Brooklyn; North Carolina—C. A. Bland, Charlotte; Ohio—L. P. Bethel, Columbus; Oklahoma—G. L. White, Oklahoma City; Oregon—George H. Nottage, Portland: Pennsylvania—Howard E. Roberts, 1515 Locust St., Philadelphia; Rhode Island—Albert L. Midgley, 312 Butler Exchange, Providence; South Carolina—L. P. Dotterer, Charleston; South Dakota—C. S. Collins, Velmillion; Tennessee—Justin D. Towner, Memphis; Texas—Rufus W. Carroll, Beaumont; Utah—W. G. Dalyrymple, Ogden; Vermont—K. L. Cleaves, Montpelier; Virginia—Wm. Pilcher, Petersburg; Washington—F. J. Shaw, Seattle, Burke Block; Wisconsin—W. H. Mueller, Madison: Mexico—Ricardo Figueroa, 1 Calle de Santo Domingo No. 8 City of Mexico. Canada—Theodore C. Trigger, St. Thomas, Ontario; Hawaii—E. L. Hutchinson, Honolulu; West Virginia—H. Bartlett, Parkersburg.

---

MICHIGAN STATE DENTAL ASSOCIATION: The annual meeting will be held June 4th and 5th in Saginaw. This is the first meeting in the second half of a century of the Society's existence and the dentists of Saginaw have determined that for cordial hospitality it shall set the pace for future sessions and places of meeting. The program is already well under way and promises to be one of exceptional interest in its literary and clinical features. All ethical practitioners will be welcomed to all privileges. It is expected that the subject of dentistry and reorganizing the State and Societies work will be completed at this meeting.

—✦—

Florida State Board of Dental Examiners: The Florida State Board of Dental Examiners will meet June 3rd., 1907, at 10 o'clock, in Jacksonville, Fla., for the purpose of examining applicants for license to practice in this State.

In addition to the written examination, applicants will be required to put in one gold filling, one alloy filling, and solder and finish 1 four-tooth bridge under supervision of Board. Bring bridge ready for investing.

Only graduates of reputable Dental schools are admitted to examination.

W. G. Mason, D. D. S.
Tampa, Fla. Sec'y of Board

—✦—

Southwestern Michigan Dental Society: This wide awake society held one of its characteristic meetings at Battle Creek April 9th and 10th. There was a large attendance of enthusiastic practitioners. The accomodations for the meeting could not have been better and helped materially to make a most pleasant and profitable two days session. The genial and generous Sherman Fowler at the head of all the Battle Creek dentists did all that could be done to make every one feel that he was glad to be there. The papers were timely and well written, and the clinics more interesting than in many larger societies.

A banquet at the elegant Post Tavern, a theater party and a visit to the large manufacturing plant of one of Battle Creek's famous food industries constituted the hosts special features in entertainment. We have'nt space to comment on these three events and could not sompare them as all three were most interesting and enjoyable. This society has a genious of its own for doing things that please one, and make him feel that the time spent in attendance was not lost, and that makes him resolve that he will always attend its meetings. The meeting next year will be held at Jackson. This is gradually coming east and it may not be long before the name will need be changed to Southern Michigan Dental Society There were 125 dentists in attendance.

—✦—

OFFICERS SOUTHWESTERN MICHIGAN DENTAL SOCIETY. For the next year are; President, O. E. Lanphear of Paw Paw; Vice President, James B. Doyle, of Grand Rapids; Secretary and Treasurer, C. W. Johnson, of Lawton.

—✦—

OHIO STATE BOARD OF DENTAL EXAMINERS: The regular semi-annual meeting of the Board of Dental Examiners of the State of Ohio will be held in Columbus June 25, 26 and 27th, 1907.

Only graduates are eligible to examination.

Application, accompanied by fee ($20.00), should be filed with the Secretary by June 15th. For further information address H. C. Brown, Secretary,
185 East State Street, Columbus, Ohio.

—✦—

AMERICAN SOCIETY OF ORTHODONTISTS: The following have been elected to fill the unexpired term as officer in this society, caused by resignation of those duly elected to the offices at the regular annual meeting. For Vice President, Dr. C. A. Hawley of Columbus, O.; to succeed Dr. R. Summa of St. Louis; Secretary and Treasurer, Dr. F. C.

Kemple, of New York City to succeed Dr. F. S. McKay of St. Louis; Chairman of the Board of Censors, Dr. F. M. Casto of Cleveland, O., to succeed Dr. E. H. Angle, of St. Louis.

---

KENTUCKY DENTAL EXAMINERS: This Board will meet June 4th in Louisville, in the Masonic Building, at 9 A. M. Applicants must be graduates of reputable colleges and present photographs with autographs certified by the Dean of the school from which he graduated. For full particulars address Dr. J. R. Wallace, The Masonic, Louisville, Ky.

---

MICHIGAN BOARD OF EXAMINERS: The next meeting will be held at Ann Arbor, in the Dental College on May 22, 23, 24 and 25, 1907. For full particulars address Dr. E. A. Honey, Kalamazoo, Mich.

---

INDIANA STATE DENTAL ASSOCIATION: Meets in Indianapolis June 11th to 13th, 1907. State Examiners meet at same time and place.

---

NATIONAL ASSOCIATION OF DENTAL EXAMINERS: Meets in Minneapolis, July 26, 1907.

---

WEST VIRGINIA STATE BOARD OF EXAMINERS: Meets in Wheeling, June 12th, 13th and 14th, 1907. H. M. VanVoorhis, Sec'y, Morgantown, W. Va.

ESSAYISTS FOR JAMESTOWN CONVENTION: The following essayists are announced for this convention to be held at Norfolk, Va., September, 10th, 11th and 12th, 1907. Professor W. D. Miller, of Ann Arbor, Mich., "Demonstration of Preparations Relating to Wasting (so called erosion) of the Teeth." Dr. Charles L. Alexander, of Charlotte, N. C., "Gold Inlays." Dr. F. T. Van Woert, of Brooklyn, N. Y., "Is the Cemented Filling the Filling of the Future?" Dr. R. Ottolengui, of New York City, "The Angle Method in Orthodontia."

AMERICAN MEDICAL ASSOCIATION, Section on Stomatology: Meets this year in Atlantic City, June 4th to 7th.

MISSOURI STATE ASSOCIATION: Meets in Kansas City, June 4th to 6th.

NATIONAL DENTAL ASSOCIATION: Meets in Minneapolis July 30th, 1907.

NATIONAL ASSOCIATION OF DENTAL FACULTIES: Meets in Minneapolis, July 26th, 1907.

